# Metagenomic analysis reveals higher *Coriobacteriia* abundance in mare’s milk consumers

**DOI:** 10.1128/mra.00862-24

**Published:** 2024-11-05

**Authors:** Nurislam Mukhanbetzhanov, Zharkyn Jarmukhanov, Samat Kozhakhmetov, Almagul Kushugulova

**Affiliations:** 1 Center for Life Sciences, National Laboratory Astana, Nazarbayev University, Astana, Kazakhstan; Montana State University, Bozeman, Montana, USA

**Keywords:** gut microbiome, *Coriobacteriia*, metagenomics, mare's milk, Coriobacteriaceae, *Atopobium*, *Olsenella*, *Enorma*, *Collinsella*

## Abstract

Our study reveals increased gut *Coriobacteriia* among mare’s milk consumers; metagenomic analysis showed a higher prevalence of genera belonging to class *Coriobacteriia* in consumers vs non-consumers. This suggests interactions between traditional dairy practices and gut microbiome composition, indicating potential for microbiota modulation through dietary interventions.

## ANNOUNCEMENT

Mare’s milk, a traditional Central Asian staple, has unexplored impacts on the human gut microbiome. Our study employs metagenomic sequencing to provide high-resolution analysis of gut microbiomes in mare’s milk consumers vs non-consumers. This approach allows for detailed taxonomic and functional profiling, enabling the identification of specific microbial species, genes, and pathways influenced by mare’s milk consumption. Our findings reveal a significant relationship with *Coriobacteriia* proliferation, highlighting potential health implications and prebiotic properties of this traditional dairy product.

We conducted a comprehensive cross-study involving 158 participants from both urban (*n* = 38) and rural (*n* = 120) populations of Kazakhstan. Based on responses to the food questionnaire regarding mare’s milk consumption, participants were divided into two groups: those who did not consume mare’s milk at all and those who frequently consumed mare’s milk. The study adhered to strict ethical standards, obtaining approval from the National Laboratory Committee of Astana (Protocol No. 02-2022, 04/04/2022, Iorg0006963) and observing the ethical principles established by the Helsinki declaration. We extracted fecal DNA using the ZymoBIOMICS DNA Kit using methodologies recommended by the manufacturer (Cat. No.: D4300). DNA concentrations were precisely determined quantitatively using the Nanodrop 2000/2000C spectrophotometer (Thermo Fisher Scientific). The shotgun metagenomic sequencing was carried out on the Illumina Novoseq 6000 platform in Novogen (Beijing, China), creating paired-end readings 150 bp ×2. The library preparation kit for metagenomic sequencing is NEBNext️ Ultra‎‎ IIDNA Library Prep Kit (Cat No. E7645). QC was performed using Novogene’s standardized pipeline, which included DNA quality assessment, library construction, and sequencing. Libraries were quantified by real-time PCR and checked with a Bioanalyzer. Phred quality scores were used to evaluate sequencing quality, with a high percentage of bases achieving Q30. Each sample yielded 5–10 GB of data, with over 80% of reads meeting the Q30 standard. MetaPhlAn v4.0.3 was used for microbial profiling, focusing on clade-specific marker genes without full genome assembly or binning.

**Fig 1:**
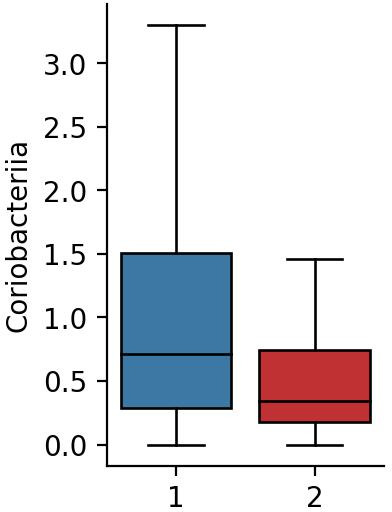
Relative abundance of class *Coriobacteriia* (*P* < 0.001): 1—mare’s milk consumers; 2—not consumers.

Our analysis revealed a significant relationship between mare’s milk consumption and an increase in the number of *Coriobacteriia* (*P* < 0.001) (Fig. 1). This conclusion confirms and expands our previous study, which demonstrated that the 60-day mode of mare’s milk consumption has led to an increase in the number of family representatives Coriobacteriaceae (1). In this study, we compared the prevalence of various genera within the class *Coriobacteriia* in raw shotgun sequencing data between mare’s milk consumers and non-consumers. Our analysis revealed the following results: *Atopobium* (0% vs 0.93%), *Olsenella* (44% vs 21%), *Enorma* (8% vsx 5.14%), and *Collinsella* (95% vs 89%).

Our study found that mare’s milk consumers were predominantly from rural populations, suggesting a potential relationship between traditional dietary practices and gut microbiome composition. The stimulation of *Coriobacteriia* growth by mare’s milk likely stems from a synergistic interplay of its unique oligosaccharides, proteins, lipids, trace elements, and physicochemical properties (2, 3).

Our groundbreaking study reveals unprecedented shifts in *Coriobacteriia* genera following mare’s milk consumption, potentially influencing inflammation, metabolism, and nutrient bioavailability (4–6). This research bridges traditional diets with microbiome science, opening avenues for probiotic interventions and highlighting the importance of preserving conventional foods as microbiome modulators.

## Data Availability

The raw sequence data from this project have been deposited under the BioProject accession number PRJNA1091163.
